# Transcriptional changes of the aging lung

**DOI:** 10.1111/acel.13969

**Published:** 2023-09-14

**Authors:** Minxue Jia, Paula A. Agudelo Garcia, Jose A. Ovando‐Ricardez, Tracy Tabib, Humberto T. Bittar, Robert A. Lafyatis, Ana L. Mora, Panayiotis V. Benos, Mauricio Rojas

**Affiliations:** ^1^ Department of Computational and Systems Biology University of Pittsburgh School of Medicine Pittsburgh Pennsylvania USA; ^2^ Joint Carnegie Mellon ‐ University of Pittsburgh Computational Biology Ph.D. Program Pittsburgh Pennsylvania USA; ^3^ Department of Internal Medicine Ohio State University Columbus Ohio USA; ^4^ Division of Rheumatology and Clinical Immunology, Department of Medicine University of Pittsburgh School of Medicine Pittsburgh Pennsylvania USA; ^5^ Department of Epidemiology University of Florida Gainesville Florida USA

**Keywords:** aging, inflammation, lung, senescence, single‐cell RNA‐seq

## Abstract

Aging is a natural process associated with declined organ function and higher susceptibility to developing chronic diseases. A systemic single‐cell type‐based study provides a unique opportunity to understand the mechanisms behind age‐related pathologies. Here, we use single‐cell gene expression analysis comparing healthy young and aged human lungs from nonsmoker donors to investigate age‐related transcriptional changes. Our data suggest that aging has a heterogenous effect on lung cells, as some populations are more transcriptionally dynamic while others remain stable in aged individuals. We found that monocytes and alveolar macrophages were the most transcriptionally affected populations. These changes were related to inflammation and regulation of the immune response. Additionally, we calculated the LungAge score, which reveals the diversity of lung cell types during aging. Changes in DNA damage repair, fatty acid metabolism, and inflammation are essential for age prediction. Finally, we quantified the senescence score in aged lungs and found that the more biased cells toward senescence are immune and progenitor cells. Our study provides a comprehensive and systemic analysis of the molecular signatures of lung aging. Our LungAge signature can be used to predict molecular signatures of physiological aging and to detect common signatures of age‐related lung diseases.

## Graphical abstract

Single‐cell RNA‐seq analysis found that alveolar macrophages and monocytes are the most transcriptionally affected cell types in the aging lung. Additionally, immune and progenitor cells in the aged lung exhibit the highest senescence score among all cell types. LungAGe score calculations revealed that changes in DNA damage repair, fatty acid metabolism, and inflammation are essential for age prediction.

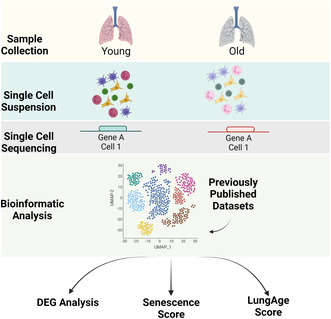

**Abbreviations**
AT1: alveolar type 1 cells
AT2: alveolar type 2 cells
CSG: cell senescence genes
DEG: differentially expressed genes
SASP: senescence associated secretory phenotype

## 1 INTRODUCTION

Aging is the natural decline of cellular function across tissues and organs, marked by decreased ability to respond to stress signals and reduced tissue reparative and regenerative capacity, increasing the risk of developing cancer and neurological, cardiovascular, and pulmonary diseases. At least nine hallmarks of aging have been identified (Lopez‐Otin et al., 2013), including epigenetic alterations, stem cell exhaustion, and cellular senescence, a state of permanent cell cycle arrest characterized by the secretion of inflammatory factors, also known as Senescence Associated Secretory Phenotype (SASP). SASP production contributes to establishing a persistent state of low and chronic inflammation, named inflammaging, which is thought to be a driving factor in developing immune senescence.

The lung is a complex organ composed of at least 61 different cell populations (Travaglini et al., 2020); single‐cell approaches are needed to capture the transcriptional changes experienced across these different cell types. Several studies have started to map the transcriptional changes of the human lung at a single‐cell level, including healthy and diseased lungs (Sikkema et al., 2023). However, many of these reports neglect to consider how age itself may be responsible for the observed outcome, or how age‐associated changes are responsible for disease development. Given the cellular complexity of the lung and that the prevalence of lung diseases increases with age, a systemic analysis of healthy tissues is needed to understand how different cell types are affected during the aging process at the transcriptional level, which can then guide therapeutic strategies. Furthermore, at a cellular level, only limited knowledge of the factors that define healthy aging of different lung cell types is available.

The multifactorial nature of aging and the complex structure of the lung have challenged our understanding of how this process occurs on a cell‐type basis in humans. In this study, we aimed to analyze the transcriptional changes at a single‐cell level in the lungs of young and aged healthy human individuals. Our analysis highlights immune senescence and senescence of stem cells, as an important component of the aging process in the lung. We also defined a score of young and aged lungs, LungAge, which reveals that aging has heterogeneous effects across cell types. Our score is a powerful tool that can predict physiological lung aging features and signatures of age‐related lung diseases. Our study fills an existing knowledge gap and provides a rich resource for future research.

## 2 METHODS

### 2.1 Preparation of frozen normal lung tissue cell suspensions and sequencing

Healthy control lungs were obtained under a protocol approved by the University of Pittsburgh, Committee for Oversight of Research and Clinical Training Involving Decedents, following rejection as candidate donors for transplant. Lung tissue was mechanically dissociated using scissors then gentleMACSTM Dissociator (Miltenyi Biotec) in an enzymatic buffer (DMEM with 10% Bovine Serum Albumin, 5 mg/mL of collagenase, 1 mg/mL of DNAse I). Samples were then passed sequentially through 100, 70, and 40 mm strainers (Flomi). Red cell lysis was completed before counting cells on Cellometer Auto 2000 using AOPI dye to assess viability (Nexcelom Bioscience). Finally, samples were frozen in 90% Fetal Bovine Serum (FBS) and 10% Dimethyl Sulfoxide. Cell suspensions were removed from liquid nitrogen, thawed using a ThawSTAR Automated Cell Thawing System (BioCision), and immediately placed in warm DMEM with 20% FBS. Cells again were counted, and viability reassessed by mixing equal volumes of cell suspension and AOPI staining solution (ViaStain, Nexelcom) were and loading into the Cell counting chamber (SD100) of the Cellometer Auto 2000 instrument (Nexcelom). The instrument automatically calculated live cells (fluorescing green) and dead cells (fluorescing red). An aliquot of 2 million cells per sample was allocated into a 2 mL DNA LoBind Eppendorf tube. Cells were then centrifuged and resuspended into a volume of 100 μL of DMEM +20% FBS. To this, 1 microliter of a unique BioLegend TotalSeq‐C cell hashing antibody was separately added to each of the samples' cells. Cells were gently mixed, incubated at 4° for 30 min, and then washed three times with 1 mL of DMEM +20% FBS. Finally, cells were filtered through a 40 mm strainer, cells were again counted, and 3500 cells from each sample were added to the master mix (10× Genomics) to form the final pool of 10 hashed samples in one 5Prime reaction well (10× Genomics). Sequencing was carried out as described previously (Morse et al., 2019; Valenzi et al., 2019).

### 2.2 Single‐cell RNA sequencing lung parenchyma dataset selection

We examined the publicly available datasets from Human Lung Cell Atlas (HLCA) (Sikkema et al., 2023) to supplement our 10 new samples, from that dataset, we filtered out: (1) samples with various lung pathologies (kept only the samples designated as “healthy”), (2) samples with smoking history (to reduce chronic lung pathologies due to smoking), (3) sample types other than “donor lung”, (4) datasets that did not provide age or smoking status information, and (5) datasets with <4 samples. The remaining datasets are, (Adams et al., 2020; Habermann et al., 2020; Reyfman et al., 2019), and samples from previous studies of ours (Morse et al., 2019; Valenzi et al., 2019). We decided to exclude the Kaminski and Banovich datasets from the primary analysis and use them for validation in the LungAge predictor. To the remaining publicly available samples that meet our criteria, we added 6 samples from our previous studies that were not included in HLCA and 10 new healthy samples that we collected data as part of this study.

### 2.3 Single‐cell RNA sequencing data analysis

Seurat package v. 4.1.0 was used for downstream analysis of unique molecular identifier (UMI) count matrices. The cell‐UMI matrix was filtered with cutoffs for cells with a number of detected gene features greater than 200, respectively, and mitochondrial percentage <35%. Empty droplets and doublets were identified and filtered out (EmptyDrops method from the DropletUtils package v.1.14.2 and Scrublet package) (Lun et al., 2019; Wolock et al., 2019). SCTransform was used for normalization, variance stabilization, data scaling, and highly variable gene selection (Hafemeister & Satija, 2019). Mitochondrial gene content percentage was regressed out when scaling data. Harmony package v. 0.1.0 was used for batch effect correction (Korsunsky et al., 2019). Seurat standard pipeline was used to generate UMAP plots and assign clusters. We identified cell populations by examining gene markers in the associated transcriptomes. Integrating our own and publicly available data brings up batch effect issues derived from different single‐cell chemistries. We aligned all datasets using Harmony (Korsunsky et al., 2019), a popular method for integrating single‐cell datasets, which interactively clusters and corrects low‐dimensional space.

### 2.4 Differential gene expression

To properly account for pseudo‐replication bias and batch effect, counts were aggregated to sample level (“Pseudobulk” approach), and batch correction was performed (Deseq2 package v.1.34.0 and Combat‐seq method from sva package v.3.42.0) (Love et al., 2014; Zhang et al., 2020). DEGs between young and aged groups were identified using a likelihood ratio test for each cell population (FDR <0.1). Clusters with <500 cells were excluded from this analysis to ensure statistical power.

### 2.5 Aging score calculation

Genes that are consistently differentially expressed across cell types (at least 1), were used. Then the average gene expression of gene sets that were either upregulated or downregulated with age was calculated and the average expression of randomly selected genes was subtracted to assess the relationship between the aging process and the gene set in question.

### 2.6 Pathway analysis and module score calculation

IPA was utilized to determine significant canonical pathways based on DEGs. Downregulated or upregulated DEGs on at least two cell populations comprised our Up‐ and downregulated gene signatures. Transcription factor enrichment analysis was performed using the two gene signatures. Module scores were inferred from the two aging‐associated gene signatures (AddModuleScore function from Seurat). For comparison, we created module scores from two published senescence gene signatures: Consensus Senescence (DePianto et al., 2021) and CSGene (Zhao et al., 2016).

### 2.7 RF classifier

To account for the batch effect, we performed such prediction evaluation separately for PGH 5P frozen dataset (10 × 5 prime V1 chemistry & frozen) and the Kaminski_2020 dataset (10 × 3 prime V2 & frozen). For age state prediction, clusters with a cell population abundance of >200 cells from a dataset were used for age state prediction. We then stratified the dataset and split it into 90:10 training and testing data using the splitstackshape package v. 1.4.8. The genes in each module were used as input features to train an RF classifier (randomForest package v.4.7–1.1) following the three‐time repeated 10‐fold cross‐validation procedure (caret package v.6.0–92). The test set was used to assess model performance with AUROC as an evaluation metric for classification.

## 3 RESULTS

### 3.1 Analysis of the aging lung at a single‐cell resolution

**FIGURE 1:**
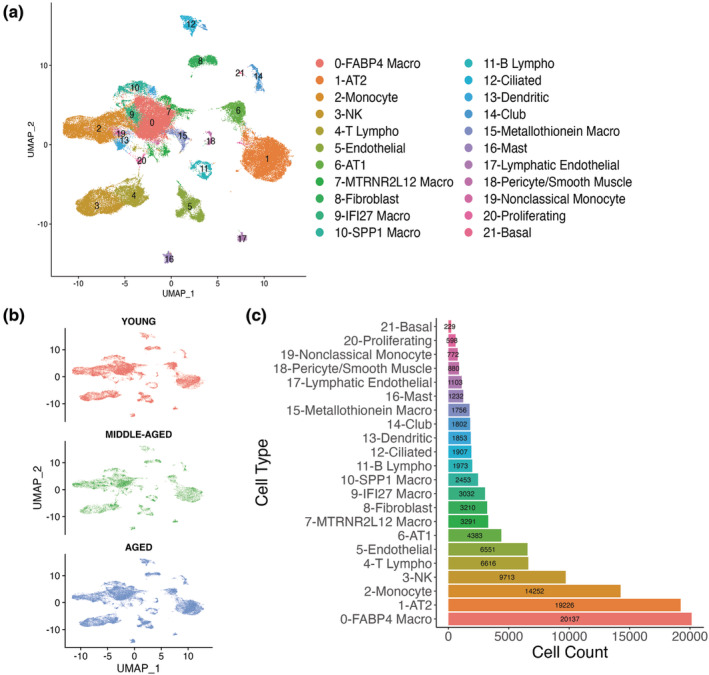
Single‐cell RNA‐Seq profiles human lung heterogeneity. (a) Uniform Manifold Approximation and Projection (UMAP) representation of the 22 identified cell populations (106,969 cells) (b) Cells on the UMAP plot of all 29 samples were colored by age group (Young: 19–23 years old, Middle‐Aged: 29–49 years old, Aged: 55–78 years old) (c) Cell counts of the identified cell types. AT1, alveolar type 1 cells; AT2, alveolar type 2 progenitor cells; lympho, lymphocytes; macro, macrophage; NK, natural killer cell.

To elucidate the transcriptional changes occurring in the lung during the aging process at the single‐cell level, we analyzed the transcriptome of lungs from 29 healthy, nonsmoker donors. These lungs were divided into three groups, young (19–23 years old), middle‐aged (29–49 years old), and aged (55–78 years old) (Table 1). A dimensionality reduction algorithm Uniform Manifold Approximation and Projection (UMAP) revealed 22 distinct clusters corresponding to the major cell types identified in the lung: stromal, endothelial, epithelial, and immune lineages (Figure 1a). Canonical marker genes used to determine cell identity can be found in Figure S1a. In our analysis, we also found two new (smaller) populations of macrophages, MTRNR2L12 Macro and IFI27 Macro, characterized by the high expression of the corresponding genes. IFI27 Macro clustered immediately adjacent to alveolar macrophages (FABP4 Macro, Figure S1c). Both macrophage clusters exhibited no differences in population abundance between young and aged individuals (Figure S2). Additionally, we also detected a cluster of proliferating cells that corresponded mainly to proliferating alveolar macrophages and proliferating T cells (Figure S3).

**TABLE 1:** Characteristics of healthy lung samples. Sample information used for cluster annotation. Further analyses were conducted in Young (≤23) versus Aged (≥55) groups.

Sample ID	Age	Gender	Chemistry	Age group	Data group	GSE	Young versus Aged
SC313	33	Male	5PrimeV1	MIDDLE‐AGED	PGH_5P	GSE212109	N
SC323	38	Male	5PrimeV1	MIDDLE‐AGED	PGH_5P	GSE212109	N
SC333	62	Male	5PrimeV1	AGED	PGH_5P	GSE212109	Y
SC334	63	Male	5PrimeV1	AGED	PGH_5P	GSE212109	Y
SC315	64	Female	5PrimeV1	AGED	PGH_5P	GSE212109	Y
HTO1	19	Female	5PrimeV1, Frozen	YOUNG	PGH_5P_Frozen	GSE150148	Y
HTO2	23	Female	5PrimeV1, Frozen	YOUNG	PGH_5P_Frozen	GSE150148	Y
HTO3	23	Male	5PrimeV1, Frozen	YOUNG	PGH_5P_Frozen	GSE150148	Y
HTO4	29	Male	5PrimeV1, Frozen	MIDDLE‐AGED	PGH_5P_Frozen	GSE150148	N
HTO6	35	Male	5PrimeV1, Frozen	MIDDLE‐AGED	PGH_5P_Frozen	GSE150148	N
HTO5	49	Female	5PrimeV1, Frozen	MIDDLE‐AGED	PGH_5P_Frozen	GSE150148	N
HTO10	55	Male	5PrimeV1, Frozen	AGED	PGH_5P_Frozen	GSE150148	Y
HTO8	70	Female	5PrimeV1, Frozen	AGED	PGH_5P_Frozen	GSE150148	Y
HTO9	74	Male	5PrimeV1, Frozen	AGED	PGH_5P_Frozen	GSE150148	Y
HTO7	78	Female	5PrimeV1, Frozen	AGED	PGH_5P_Frozen	GSE150148	Y
Donor 8	21	Male	3PrimeV2	YOUNG	CHI_V2	GSE122960	Y
Donor 6	22	Female	3PrimeV2	YOUNG	CHI_V2	GSE122960	Y
Donor 3	29	Female	3PrimeV2	MIDDLE‐AGED	CHI_V2	GSE122960	N
Donor 2	55	Male	3PrimeV2	AGED	CHI_V2	GSE122960	Y
Donor 4	57	Female	3PrimeV2	AGED	CHI_V2	GSE122960	Y
Donor 1	63	Female	3PrimeV2	AGED	CHI_V2	GSE122960	Y
SC31	56	Male	3PrimeV1	AGED	PGH_V1	GSE128033	Y
SC14	76	Male	3PrimeV1	AGED	PGH_V1	GSE128033	Y
SC59	18	Male	3PrimeV2	YOUNG	PGH_V2	GSE128033	Y
SC155	23	Female	3PrimeV2	YOUNG	PGH_V2	GSE128033	Y
SC156	23	Female	3PrimeV2	YOUNG	PGH_V2	GSE128033	Y
SC45	55	Male	3PrimeV2	AGED	PGH_V2	GSE128033	Y
SC56	57	Female	3PrimeV2	AGED	PGH_V2	GSE128033	Y
SC277	21	Male	3PrimeV3	YOUNG	PGH_V3	GSE212109	N

To determine if the cell‐type annotation was biased by age, we independently visualized cells in UMAP embedding for each age group. We found that all cell types were evenly represented in all three age groups (Figure 1b) and across batches (Figure S1b). Due to different experiment protocols, samples from the Chicago cohort (CHI_V2) exhibited different cell‐type proportions from other groups (Figure S2). Therefore, we evaluated the aging effect in each cohort containing both young and aged samples (CHI_V2, PGH_V2, PGH_5P_Frozen) and then compared the results. The age‐associated decline was noticed in monocyte and AT1 cells and increments in AT2 and basal cells (Figure S4). However, the aging‐associated change in the population abundance of those cells was not statistically significant. We also evaluated the ratio of each cell type and found that FABP4 macrophages were the most represented cluster, followed by AT2 cells, Monocytes, and NK cells (Figure 1c).

### 3.2 Aging effects on specific cell populations

**FIGURE 2:**
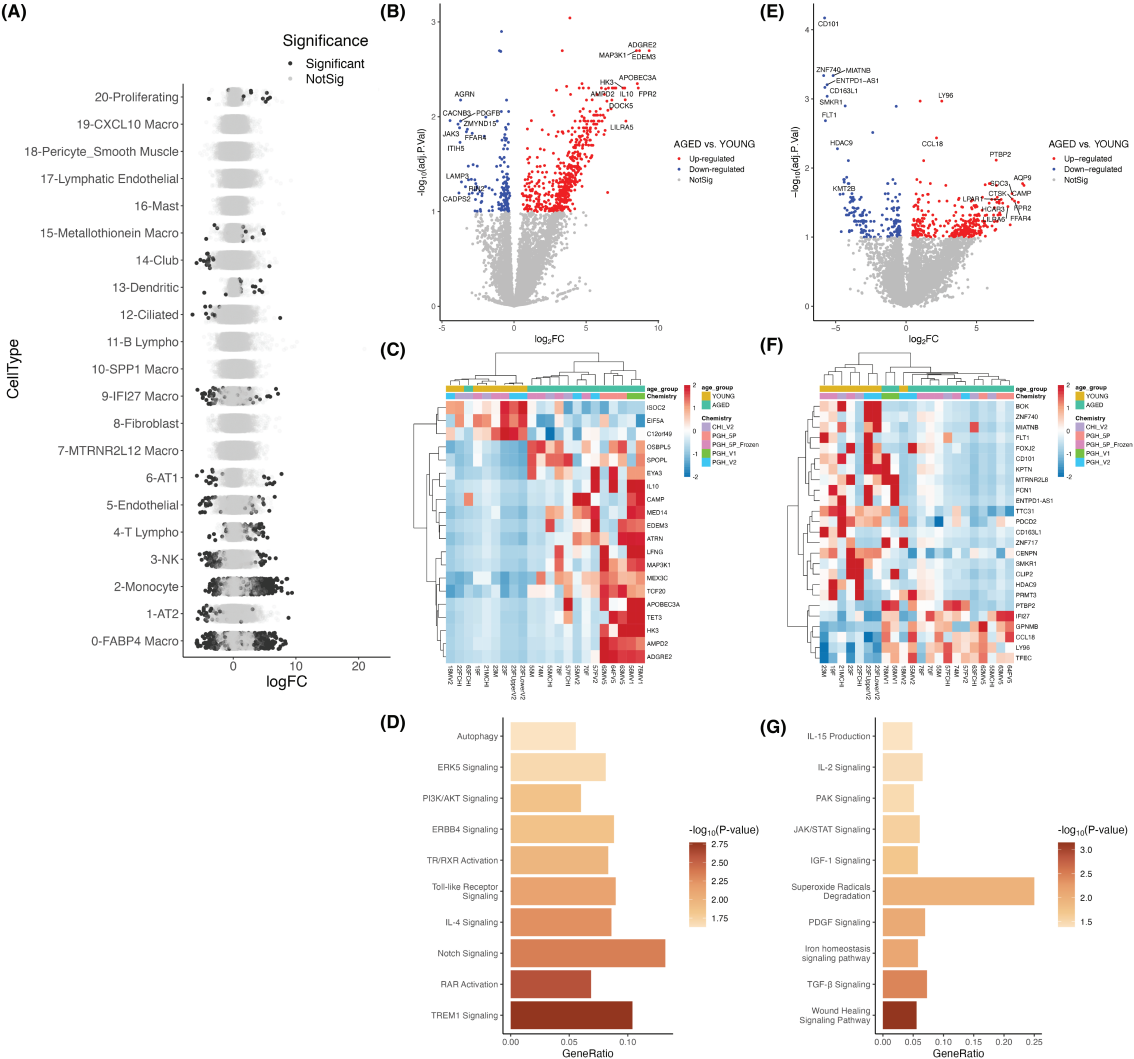
Differential expression analysis of single‐cell RNA‐Seq data on young and aged lungs identifies aging genes across cell types. (a) Differentially expressed genes (DEGs) with darker color indicating statistical significance (adjusted *p* < 0.1). Volcano plots and heatmaps (top 20 most significant DEGs) are shown for monocyte‐like (b, c) and FABP4 macrophages (e, f). Enriched canonical pathways of monocyte‐like (d) and FABP4 macrophages (g) were detected using IPA. GeneRatio is the fraction of DEGs found in the specific gene set/pathway.

Lung function is relatively stable after reaching a peak at age 20 to 25 and then starts declining at age 50 due to physiological lung aging (Agusti & Faner, 2019). Thus, we focused our subsequent analysis on the young (≤23) and aged (≥55) groups. To systematically study the influence of age on each of the 22 cell populations identified, we performed differential gene expression analysis between young and aged samples (Figure 2a). To overcome the dropout effect, the batch effect, and the imbalanced cell population, we aggregated information across cells for each individual (“Pseudobulk” method) and then corrected batch effects derived from different sequencing chemistries using Combat (Zhang et al., 2020). This also helped overcome the issue of statistical power for differentially expressed gene (DEG) detection when the number of cells per individual is small. As expected, the number of DEGs correlated with the number of cells in each cell cluster. In general, we found that age affects the transcriptional landscape at several levels. For example, fibroblasts, B lymphocytes, or SPP1/interstitial macrophages did not exhibit any significant changes between young and aged, while endothelial, AT1, and IFI27 macrophages exhibited a moderate number of genes whose expression was influenced by age. Volcano plots showing DEGs for each cell population are provided in Figure S5. We found that monocyte‐like macrophages, with 705 DEGs (FDR <0.1), were among the cell populations with the highest transcriptional changes, followed by FABP4 macrophages with a total of 498 DEGs. Interestingly, monocytes and macrophages play a central role in initiating inflammaging, a pivotal component of the aging process that leads to compromised immune function.

Given the high influence of aging in the transcriptome of monocytes and alveolar macrophages, we focused our subsequent analysis on these two clusters. When we looked at the transcriptional changes in monocyte‐like macrophages, we found 552 upregulated and 153 downregulated genes (FDR <0.1) (Figure 2b,c). We saw a significant upregulation of proinflammatory genes ADGRE2, APOBEC3A, and LILRA5 and an upregulation of IL‐10. In addition to inflammation‐related genes, we found that aged monocyte‐like macrophages overexpressed cellular stress genes such as EDEM3 and MAP3K1. Altogether, upregulation of these genes, links aging to inflammation and activation of stress responses in aged monocytes.

Among the genes whose expression was downregulated in monocyte‐like macrophages, we found AGRN, PDGFB, and tyrosine kinase JAK3. These genes are critical for monocyte function and interaction with other cell types involved in tissue repair (Mazzon et al., 2012; Pierce et al., 1991). By performing ingenuity pathway analysis (IPA) (Figure 2d), we found an enrichment of TREM1 signaling; this pathway plays an essential role in tissue repair processes and regulates the ER stress response, ROS production, and the Toll‐like Receptor (TLR)‐mediated inflammation. Interestingly, we also found TLR signaling enrichment. Enrichment of TREM1 and TLR pathways in aged monocytes suggests that these two processes synergize to produce proinflammatory molecules contributing to the inflammaging process. We also found that Retinoic Acid Receptor (RAR) activation was enriched in the aged group. RAR activation influences monocyte to macrophage differentiation, and enhances the inflammasome response in macrophages; additionally, this pathway has been associated with the senescence process by regulating calcium signaling. Other pathways associated with senescence (Notch and IL‐4 signaling) were also enriched in aged monocytes.

FABP4 Macrophages represent most of the alveolar macrophage population (Morse et al., 2019). To better understand how age affects the transcriptional landscape in alveolar macrophages (Figure 2e,f), we found that the most downregulated gene in the aged population was CD101, a critical factor for the production of anti‐inflammatory cytokines (Schey et al., 2016), followed by the transcription factor ZNF740, the long noncoding RNAs (lncRNA): MIATNB and ENTPD1‐AS1, as well as histone deacetylase 9 (HDAC9) and lysine methyltransferase 2B (KMT2B). Among the upregulated genes in aged macrophages, we found cathelicidin antimicrobial peptide (CAMP), an essential mediator of inflammatory responses, cell surface proteoglycan SDC3, associated with inflammation and wound healing, and we also found upregulation of genes related to metabolic homeostasis like HCAR3 and FFAR4. We performed an IPA analysis to better understand the pathways affected in alveolar macrophages during aging (Figure 2g). We found increased wound healing, IL‐2 signaling, IL‐15 production, and iron homeostasis. These pathways are associated with macrophage function and inflammation (Nelson et al., 1994; Perera et al., 2012; Winn et al., 2020) and are associated with the aging process.

### 3.3 Identification of lung aging (LungAge) signatures

**FIGURE 3:**
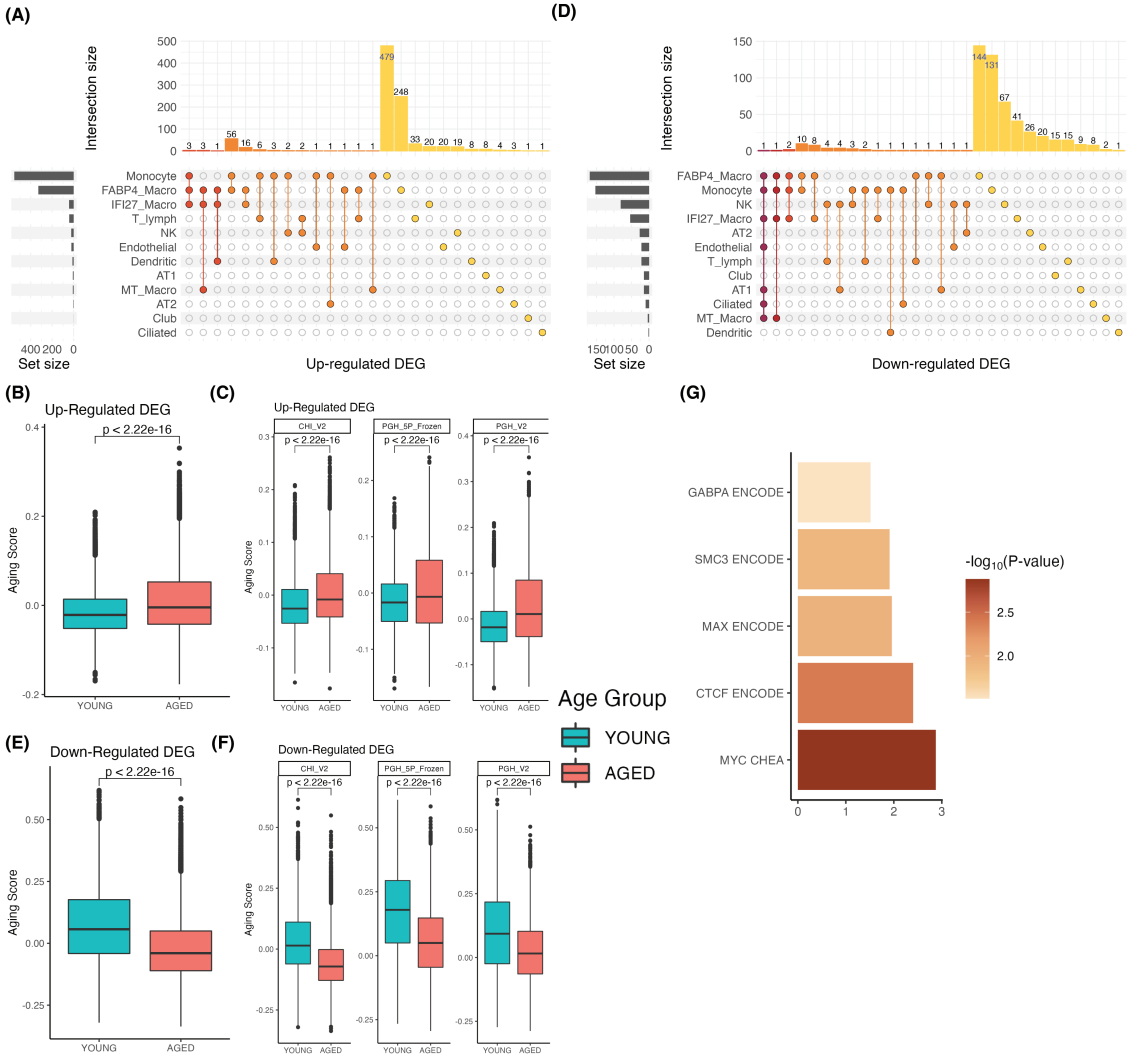
Identification of Lung Aging gene signatures. Upset plots to visualize significant upregulated (a) and downregulated (d) differentially expressed genes (DEGs). In upset graphs, each column corresponds to an intersection set. Bar charts (top) show the size of the set. Colors indicate the number of cell types in the intersection set from red (7 cell types in d) to yellow (one cell type). Each row represents which intersection sets each cell type participates. (b) Module aging scores of all cell types and cohorts were inferred based on upregulated DEG set (Wilcoxon test). (c) Breakdown by cohort of the results in (b). (e) Module aging scores of all cell types and cohorts were inferred based on the downregulated DEG set. (f) Breakdown by the cohort of the results in (e). (g) Enrichr detected enriched transcription factors of downregulated gene signature.

Next, we identified general lung aging gene signatures by summarizing those genes that are consistently DEGs across cell types. These signatures were defined separately for up‐ and downregulated genes, then module scores (gene set activities) were calculated as the average gene expression of gene sets that were either upregulated or downregulated with age and subtracting the average expression of randomly selected genes. This way, we can assess the relationship between the aging process and the gene set in question. Figure 3a presents an upset plot where the number of upregulated genes in each cell type can be visualized. Each column represents a set of genes that are significantly upregulated in one or more cell types, and the color of each column represents the number of cell types where such genes were upregulated (red indicates the highest number of cell types and yellow the lowest one). For example, the first column corresponds to three genes that are consistently upregulated with age in monocytes, FABP4 macrophage, and IFI27 macrophage cells. Our upregulated gene aging score is based on the expression of all 97 upregulated genes found in two or more cell types (Figure 3a, dark orange). The distribution of this score in young and aged cells is presented in the boxplots of Figure 3b,c.

We then calculated the upregulated score for all cell types identified in our analysis, aiming to identify what cell populations of the lung are transcriptionally more vulnerable to aging than others based on a higher score (Figure S6). We found that overall, cells from aged lungs exhibited a higher upregulated gene score than cells from the young controls, except for club, mast, and basal cells, which exhibited no significant differences. Among the cells with the most significant *p*‐value, we found cells from the immune lineage, including 5 clusters of macrophages, dendritic cells, monocytes, and T lymphocytes, together with AT2 cells and endothelial cells (Figure S6). Interestingly, most of the cells with a significantly higher score were either immune or progenitor cells, an observation that has been made in other tissues (Zhang et al., 2021).

A similar analysis was done with the downregulated genes (Figure 3d–f). We found that most of these 43 genes were represented in the FABP4 Macrophages cluster. The maximum number of clusters where a particular gene was downregulated was 7 (Figure 3d). As expected, the aged group exhibited a significantly lower score than the young controls (Figure 3e), which was represented across the three groups of samples (Figure 3f). Looking at the downregulated score on an individual cell‐type basis (Figure S7), we found that immune cells, AT2, AT1, and endothelial and club cells exhibited a lower score in the aged group compared to young controls. This result correlates with the higher upregulated signature observed in some cell types.

Enrichment analysis showed that the upregulated signature was enriched for the CCCTC‐binding factor (CTCF) (*p* < 0.05). CTCF is a zinc finger transcription factor that can activate or repress gene expression and influences chromatin organization. Interestingly, this transcription factor is enriched in both upregulated and downregulated gene sets (Figure 3g), highlighting its role in transcriptional regulation during aging. Given that CTCF modulates chromatin topology and that alterations of chromatin structure have been associated with aging, this result indicates that lung cells may experience chromatin reorganization during aging, most likely inducing the transcriptional changes observed in our analysis. Strikingly, we found a higher number of transcription factors enriched in the downregulated gene set. Among these, besides CTCF, we found MYC, MAX, SMC3, and GABPA; these transcription factors are associated with proliferation, DNA repair, regulation of metabolism, cell cycle, and protein synthesis (Guney et al., 2006; Hydbring & Larsson, 2010), all these are biological processes affected by age.

### 3.4 The LungAge classifier achieves good performance on aging state prediction

**FIGURE 4:**
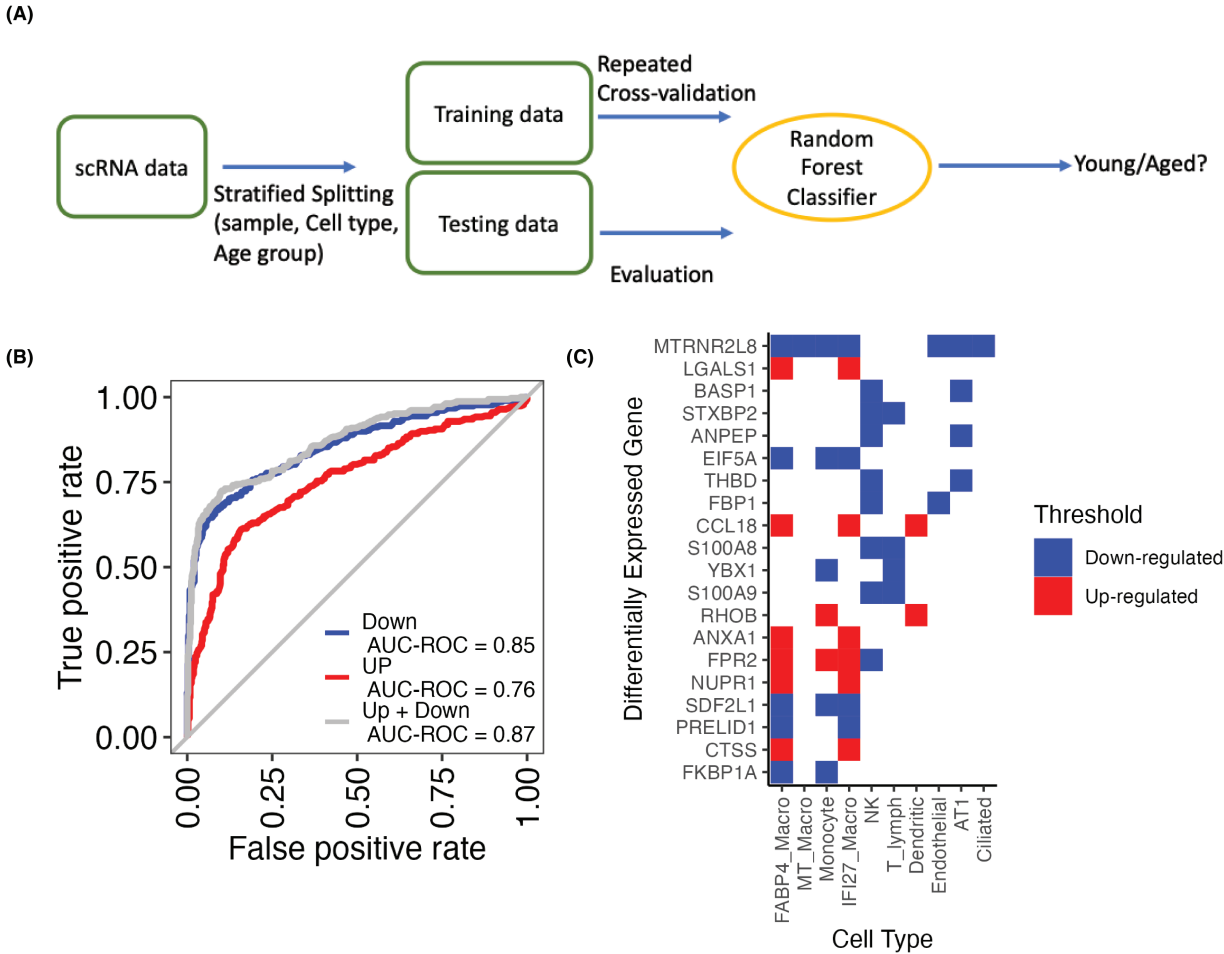
Random forest (RF) classifier for age state prediction. (a) The Lung Aging Signature gene features of the PGH 5P Frozen dataset with age group annotation (label) were randomly stratified into training data and testing data by sample, cell type and age group. A RF classifier was trained with the training data to predict the aging state (Young/Aged). (b) The area under the receiver operating characteristic (AUROC) evaluated prediction power of the RF classifier on the testing data using upregulated differentially expressed genes (DEGs) only (Red), downregulated genes only (Blue), and all DEGs (Grey). (c) Heatmap of the top 20 most important variables in the classification task colored as either upregulated in red or downregulated in blue across cell types.

To validate the discriminatory power of our LungAge gene signatures, we built a random forest (RF) classifier based on LungAge genes to predict the aging state (Figure 4a). The PGH 5P Frozen dataset (10 × 5 prime V1 chemistry, frozen), including cells from lung tissues of 3 young and 4 aged samples, was used to train and evaluate the classifier. To systematically assess aging‐associated genes, we randomly stratified input dataset by sample, cell type, and age group, and then used a repeated 10‐fold cross‐validation procedure for model training. The classifier achieved good performance on the prediction task (area under the receiver operating characteristic [AUROC]: 0.87) (Figure 4b). Interestingly, downregulated genes are more informative in delineating the aging of human lung tissue (AUROC: 0.85) than upregulated genes (AUROC: 0.76). Among the top 20 genes with the highest variable importance for our prediction task, we found proinflammatory genes (S100A8 and S100A9), genes associated with DNA damage repair (YBX1), fatty acid metabolic genes, transcriptional regulator (NUPR1), and CCL18, a surrogate gene for the expression of IL‐6 which increases with aging (Figure 4c). These top 20 genes are differentially expressed in 10 out of the 22 cell types identified, including immune populations FABP4 Macro, IFI27 Macro, and NK cells. We conducted further validation of the information value of our LungAge gene signatures by analyzing scRNA‐seq profiles of lungs from healthy nonsmokers in two datasets (Table 2): (Adams et al., 2020; Habermann et al., 2020). To evaluate the effectiveness of the RF classifier trained using the PGH 5P Frozen dataset, we assessed it with 7 old samples (ages 55–69, 10 × 5 prime V1 chemistry, fresh) from the Habermann et al. (2020) dataset (Habermann et al., 2020). The results showed that most cells were correctly classified, with an accuracy of 0.87. To avoid potential batch effect in scRNA‐seq data, another RF classifier was trained for the Adams et al. (2020) dataset (4 young and 8 old samples, 10 × 3 prime V2, frozen). The results were found to be essentially the same (Figure S8).

**TABLE 2:** Information of healthy nonsmoker lung parenchyma samples used for aging state classifier validation.

Sample ID	Age	Sex	Chemistry	Age group	Fresh or frozen	Ever smoker	DOI
VUHD103	55	Male	5PrimeV1	AGED	fresh	N	https://doi.org/10.1101/2022.03.10.483747
VUHD92	55	Male	5PrimeV1	AGED	fresh	N	https://doi.org/10.1101/2022.03.10.483747
VUHD105	58	Male	5PrimeV1	AGED	fresh	N	https://doi.org/10.1101/2022.03.10.483747
VUHD94	62	Male	5PrimeV1	AGED	fresh	N	https://doi.org/10.1101/2022.03.10.483747
THD0014	64	Male	5PrimeV1	AGED	fresh	N	https://doi.org/10.1101/2022.03.10.483747
VUHD95	64	Female	5PrimeV1	AGED	fresh	N	https://doi.org/10.1101/2022.03.10.483747
VUHD98	69	Male	5PrimeV1	AGED	fresh	N	https://doi.org/10.1101/2022.03.10.483747
081C	20	Male	3PrimeV2	YOUNG	Frozen	N	https://doi.org/10.1126/sciadv.aba1983
1372C	21	Female	3PrimeV2	YOUNG	Frozen	N	https://doi.org/10.1126/sciadv.aba1983
001C	22	Male	3PrimeV2	YOUNG	Frozen	N	https://doi.org/10.1126/sciadv.aba1983
208C	23	Male	3PrimeV2	YOUNG	Frozen	N	https://doi.org/10.1126/sciadv.aba1983
465C	56	Male	3PrimeV2	AGED	Frozen	N	https://doi.org/10.1126/sciadv.aba1983
388C	61	Male	3PrimeV2	AGED	Frozen	N	https://doi.org/10.1126/sciadv.aba1983
192C	62	Female	3PrimeV2	AGED	Frozen	N	https://doi.org/10.1126/sciadv.aba1983
160C	64	Male	3PrimeV2	AGED	Frozen	N	https://doi.org/10.1126/sciadv.aba1983
439C	66	Female	3PrimeV2	AGED	Frozen	N	https://doi.org/10.1126/sciadv.aba1983
065C	66	Female	3PrimeV2	AGED	Frozen	N	https://doi.org/10.1126/sciadv.aba1983
003C	67	Female	3PrimeV2	AGED	Frozen	N	https://doi.org/10.1126/sciadv.aba1983
296C	80	Female	3PrimeV2	AGED	Frozen	N	https://doi.org/10.1126/sciadv.aba1983

### 3.5 Senescence scores for the aging lung

To determine if the transcriptional changes observed in the aged lung are associated with increased senescence, we calculated the senescence score of young and aged lungs employing two previously published gene sets: Consensus Senescence and CSGene. The first set corresponds to 11 genes shared by senescent lung fibroblasts and senescent lung epithelial cells (*Consensus Senescence*) (DePianto et al., 2021). The second consists of the top 20 ranked cell senescence genes (*CSGene*) (Zhao et al., 2016). We first interrogated what proportion of genes identified in our analysis was represented in the two senescence gene sets and found no overlap with the upregulated or downregulated genes list (Figure S9a,b). We used each of these two published gene sets to calculate the senescence score of young and aged lung samples. We found that the Consensus Senescence (Figure S9c), the CSGene (Figure S9d), or the union of the two (Figure S9e) exhibited a significantly higher score in aged samples than the young controls. This higher senescence score was observed across the three different groups of samples analyzed (Figure S9f–h), except for the PHG_V2 cohort that yielded no significant differences between aged and young controls for the CSGene set (Figure S9g).

**FIGURE 5:**
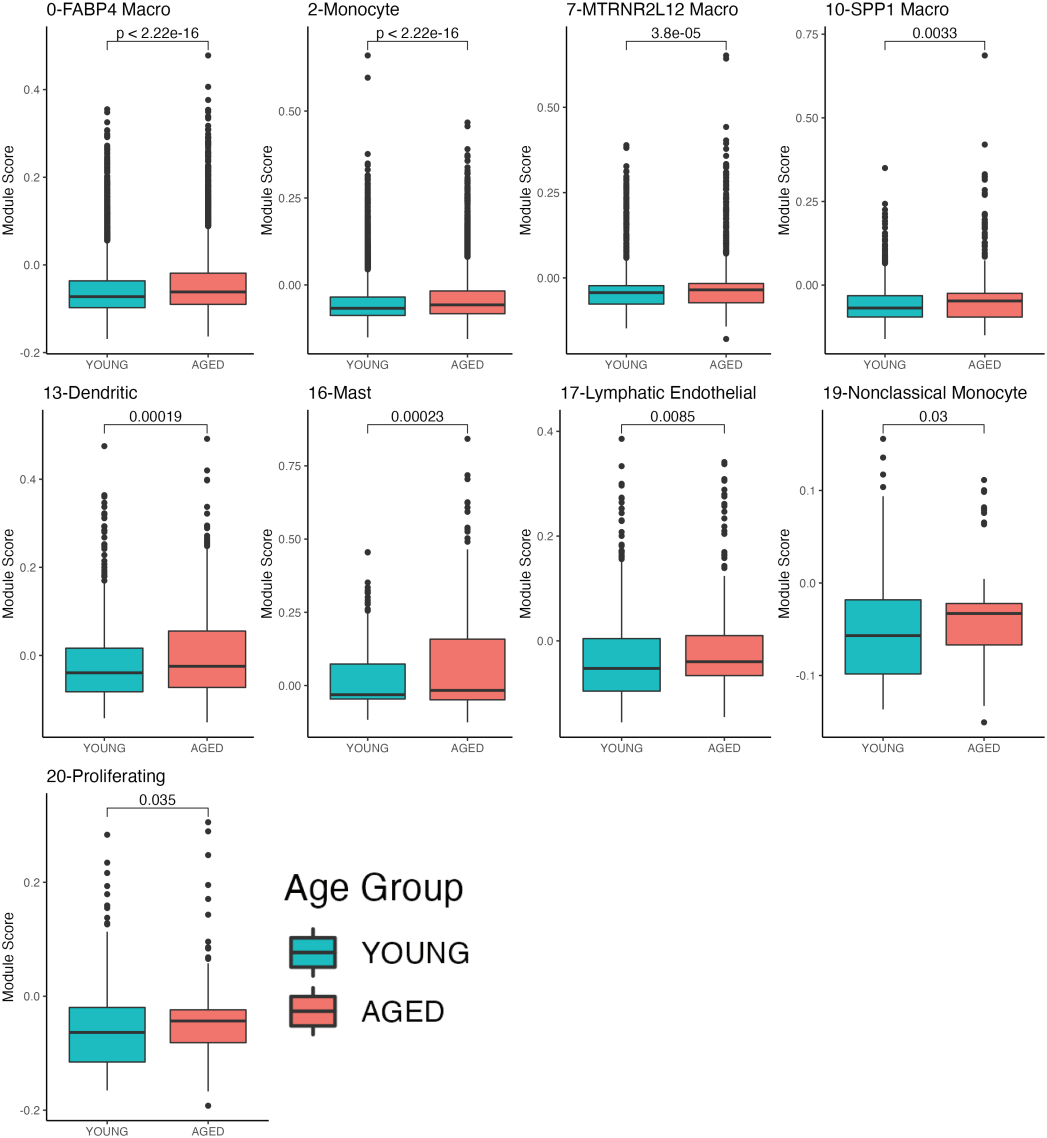
Specific cell populations exhibit a higher score distribution of consensus senescence gene signature. Boxplots of module score distribution of Consensus Senescence gene signature in cell populations. Only cell types with significant differences are presented (Wilcoxon test).

**FIGURE 6:**
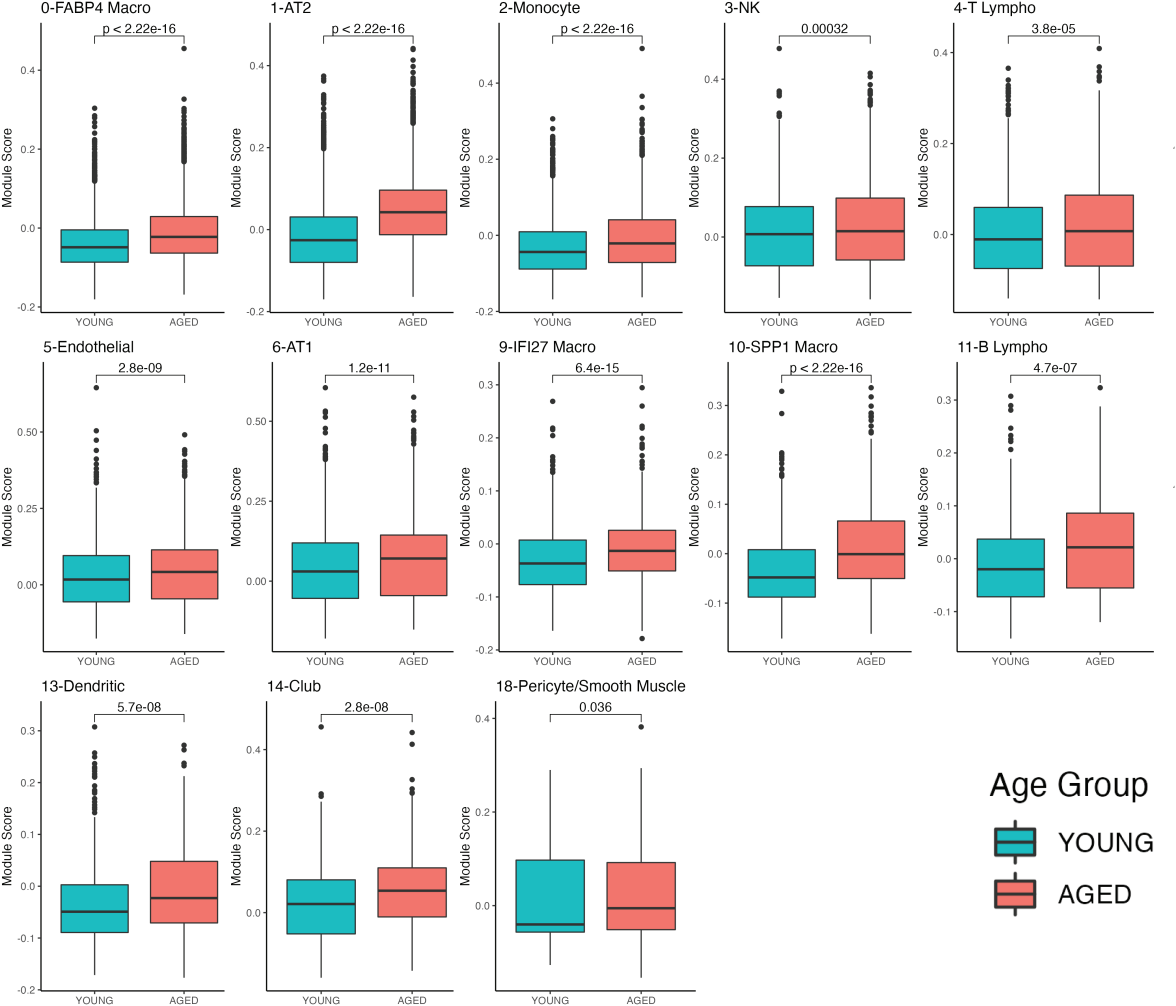
Specific cell populations exhibit a higher score distribution of CSGene signature. Boxplots of module score distribution of CSGene gene signature in cell populations. Only cell types with significant differences are presented (Wilcoxon test).

Since increased senescence in specific cell types has been associated with chronic lung diseases, we evaluated the senescent state of each cell population identified in our analysis using the Consensus Senescence and the CSGene sets. We found that certain cell types are more biased toward senescence than others. For example, when using the Consensus Senescence gene set, we found that cells with a significantly higher score in the aged lungs were mostly immune cells (FABP4 macro, monocytes, MTRNR2L12 macro, Spp1 macro, dendritic, mast, lymphatic endothelial, nonclassical monocyte cells) in addition to proliferating cells (Figure 5). Although the fact that the proliferating cluster exhibited a higher senescence score may seem counterintuitive, we noted that this cluster contained mainly markers of immune cells (Figure S3), pointing to the history of immune responses in aged donors and supporting our observation that immune populations are more biased toward senescence in the aged lung (Figure 5). When we calculated the senescence score using the CSGene set we found congruency in a higher senescence score for FABP4 macro, monocytes, SPP1 macro, and dendritic cells. We also found a significantly higher senescence score of the aged group on epithelial (AT2, AT1, club), stromal (pericyte/smooth muscle, endothelial), and lymphoid cells (NK, T, and B lymphocytes and IFI27 macro cells) (Figure 6).

Interestingly, using the CSGene set yielded a higher senescence score in AT2 and club cells, two cell types with stem cell potential. These results were further corroborated by combining the two gene sets (Figure S10). Our senescence scores highlight immune senescence and senescence of stem cells, as an important component of the aging process in the lung.

## 4 DISCUSSION

Our analysis successfully identified 22 cell types and found that aging influences different cellular populations in unique ways, as some of them acquire a distinct transcriptional signature while others remain transcriptionally stable during aging. For example, we did not observe age‐associated changes in B lymphocytes, fibroblasts, or smooth muscle cells. On the contrary, the effect of aging was transcriptionally more severe on monocyte‐like and alveolar macrophages; this might be due to a much larger number of these cells detected than other cell types increasing the power to identify differences. Monocyte‐like macrophages exhibited upregulation of IL‐10. Interestingly, IL‐10‐producing cells accumulate during aging, leading to age‐associated immune suppression and immune senescence (Almanan et al., 2020). And it has been proposed as a biomarker of physiological aging (Sanchez‐Molina et al., 2022).

Recent studies have highlighted the lung epithelium as a central player in the repair process of acute and chronic injuries, a process impaired in the elderly. We found in our analysis that although alveolar epithelial type 2 (AT2) cells exhibited a low number of DEGs between young and old individuals. These DEGs included EP300, TOX3, and ETV2. Interestingly, another member of the ETV family, ETV5 is essential for the maintenance of the AT2 cells and to efficiently repair the mouse lung following bleomycin‐induced injury (Zhang et al., 2017). However, the function of ETV2 in the lung epithelium has yet to be reported. The miss regulation of the transcriptional regulators EP300, TOX3, and ETV5, suggests that AT2 cells from old individuals undergo chromatin changes, that can compromise their capacity to engage into differentiation programs, affecting the elderly the capacity to repair lung injuries. Furthermore, it has been shown that p300 induces the formation of de novo super‐enhancers that regulate the senescence process (Sen et al., 2019), accumulation of senescent AT2 cells is a driver of pulmonary fibrosis (Yao et al., 2021). These data suggest that EP300 overexpression in old AT2 cells leads to the establishment of senescence programs that promote the development of lung fibrosis.

Identifying age‐related signatures in the lung is an essential tool to better understand how specific cell populations respond to aging. Here, we have calculated LungAge scores, which can be helpful in the prediction of symptoms of physiological aging and the identification of common factors among age‐related diseases. Using the LungAge signatures in a prediction task, we found that the most highly predictive variables were associated with DNA damage repair, inflammation, and fatty acid metabolism (YBX1, S100A8, S100A9, and FBP1). The relationship between these three processes and aging has been widely studied. As we age, our cells accumulate DNA damage. In the case of lung cells, this event is further exacerbated by the constant exchange and exposure to the environment. Decreased or compromised DNA damage repair leads to impaired cell fate decisions affecting tissue repair; and promoting genome instability, which induces senescence, a hallmark of aging and a critical factor in the development of inflammation and age‐related diseases. In recent years, fatty acid metabolism has gained importance as a central modulator of aging and lifespan, where fatty acid enzymes significantly change with age and influence the development of age‐related pathologies (Mutlu et al., 2021). A tight correlation between DNA damage, aging, and lipid metabolism has been made in other organisms (Hamsanathan et al., 2022), where extensive DNA damage drives aging through the misregulation of fatty acid metabolism and upregulation of genes associated with the innate immune response. An exciting possibility is that in lung cells, accumulation of DNA damage during our lives, promotes changes in fatty acid metabolism, influencing the expression of a proinflammatory signature leading to aging and priming the tissue for the development of age‐related diseases. CCL18 was among the most critical predictors of lung aging, this chemokine is associated with pulmonary fibrotic activity (Prasse et al., 2007), and its increased expression is most likely reflecting increased levels of IL‐6 (Khanna et al., 2016), a central component of SASP that has been implicated in pulmonary inflammatory diseases.

Transcription factors are essential for establishing transcriptional signatures in both health and disease. Additionally, this group of proteins represents a direct therapeutic target in diseases like cancer. Identifying the transcription factors that regulate aging in the lung represents a crucial therapeutic avenue in the case of chronic age‐related diseases. Using our LungAge gene signature, we found that CTCF regulates both the upregulated and the downregulated gene signatures. Our results agree with the AgeAnno study, which used scATAC‐seq data from human lungs and found that CTCF is altered on fibroblast and epithelial cells during aging (Huang et al., 2023). Although it may seem contradictory, CTCF is known to act as both a transcriptional repressor and activator and a chromatin insulator that blocks the interactions between regulatory elements, hence influencing gene expression and chromatin organization (Kim et al., 2015). CTCF has been associated with mediating replicative senescence through its interactions with DNA polymerase (Hou et al., 2021) and the induction of an inflammatory signature in senescence and cancer (Miyata et al., 2021). MYC, a transcription factor known for moonlighting as an oncogene in certain cancers, has been reported to repress senescence in several scenarios. It has also been shown that its activity can promote proliferation and differentiation in aged progenitor cells (Neumann et al., 2021). SMC3, also found in the downregulated signature, is a subunit of the cohesin complex, involved in chromatin organization, transcriptional regulation, and DNA damage repair. Besides the previously described relationship between chromatin organization and aging, accumulation of DNA damage has been proposed as an early event during senescence, suggesting that SMC3 may prevent the onset of aging hallmarks. Interestingly GABPA, which was also found enriched in the downregulated gene signature, binds the TERT promoter and regulates telomerase activity (Guo et al., 2020); this transcription factor is also involved in the regulation of protein synthesis, metabolism, and cell cycle control, processes that are altered with aging.

The accumulation of senescent cells is a hallmark of aging and an important factor in developing age‐related diseases; in the lung, increased cellular senescence leads to compromised regenerative capacity and, ultimately, to the development of fibrosis. The identification of senescent cells in tissues has been challenging as different triggers of senescence and particular cell types exhibit specific senescence features. Here we used two different gene sets previously published to identify and quantify senescence in the lung on a cell‐type basis. We found that a higher upregulated LungAge score is not strictly correlated with a higher senescence score, indicating that not all aged cells will necessarily become senescent. Additionally, a higher senescent score did not directly correlate either with transcriptional dynamics, as cells that were transcriptionally stable during aging exhibited a high senescence score, for example, SPP1 macrophages and B lymphocytes. We found that we captured more senescent populations using the CSG gene list than the 11 consensus genes. Our data shows that lung cell populations with a higher senescence score are mostly immune or progenitor cells. Immune senescence and senescence of stem or progenitor cells are characteristic of aging and contribute to the decline in lung function and impaired tissue regeneration.

One of the biggest challenges to study aging‐associated changes in the human lung is sample collection, as normal healthy lungs are usually accepted for transplants and the rejected normal lungs may be compromised with unexpected pathologies. Here, we used samples from carefully screened donors to avoid history of smoking or any other pathological issues. All the Pittsburgh samples (including our new samples) are clear of these pathologies, and all but one of the Chicago cohort samples are from never‐smokers (one sample comes from a former smoker). This creates a limitation of our study, because the sample size may limit the number of cellular populations represented in our data for certain cell types. Additionally, the low cell number may impact the statistical power of our DEG analysis. When the abundance of sample‐wise cell‐type populations is not large enough, the statistical power for detecting DEGs may be compromised, leading to suboptimal outcomes in DEG detection. To overcome this limitation, we utilized the pseudobulk method with the sum aggregation to boost the sensitivity of DEG detection. Previous reports have shown that high environmental exposures or high pollution levels are associated with increased DNA methylation age, which can predict health status (Dhingra et al., 2018). A critical consideration in future studies is the environmental exposure to which the donors have been exposed and how it influences the transcriptional changes of the human lung here described.

Altogether our study presents, to our knowledge, the first systemic analysis of the aging human lung. We identified monocyte‐like and alveolar macrophages as the most transcriptionally dynamic cell types during aging. Finally, our LungAge signature poses an essential tool in the field, as it can be used to predict symptoms of physiological aging and to better understand the molecular pathways linking age and chronic diseases in the lung.

## AUTHOR CONTRIBUTIONS

Ana L. Mora, Robert A. Lafyatis, Panayiotis V. Benos, and Mauricio Rojas involved in conception and design: Minxue Jia, Paula A. Agudelo Garcia, Jose Antonio Ovando‐Ricardez, and Tracy Tabib provided the experimental work analysis and interpretation. Paula A. Agudelo Garcia, Minxue Jia, Ana L. Mora, Robert A. Lafyatis, Panayiotis V. Benos, and Mauricio Rojas involved in drafting the manuscript and intellectual content. All authors approved the final version of the manuscript.

## CONFLICT OF INTEREST STATEMENT

The authors state no conflict of interest.

## Supporting information


Figure S1.
Figure S2.
Figure S3.
Figure S4.
Figure S5.
Figure S6.
Figure S7.
Figure S8.
Figure S9.
Figure S10.

## Data Availability

The scRNA‐seq datasets were downloaded from GSE122960, GSE128033, GSE128169, GSE150148, GSE136831, and https://beta.fastgenomics.org/p/hlca.
