# Supplementary material for: Transcriptional changes of the aging lung
Source: Aging Cell. 2023 Sep 14;22(10):e13969. doi: 10.1111/acel.13969 (PMC10577555; doi:10.1111/acel.13969)
Supplement: Supplementary file 1 — Figure S1. Figure S2. Figure S3. Figure S4. Figure S5. Figure S6. Figure S7. Figure S8. Figure S9. Figure S10. [file ACEL-22-e13969-s001.docx]

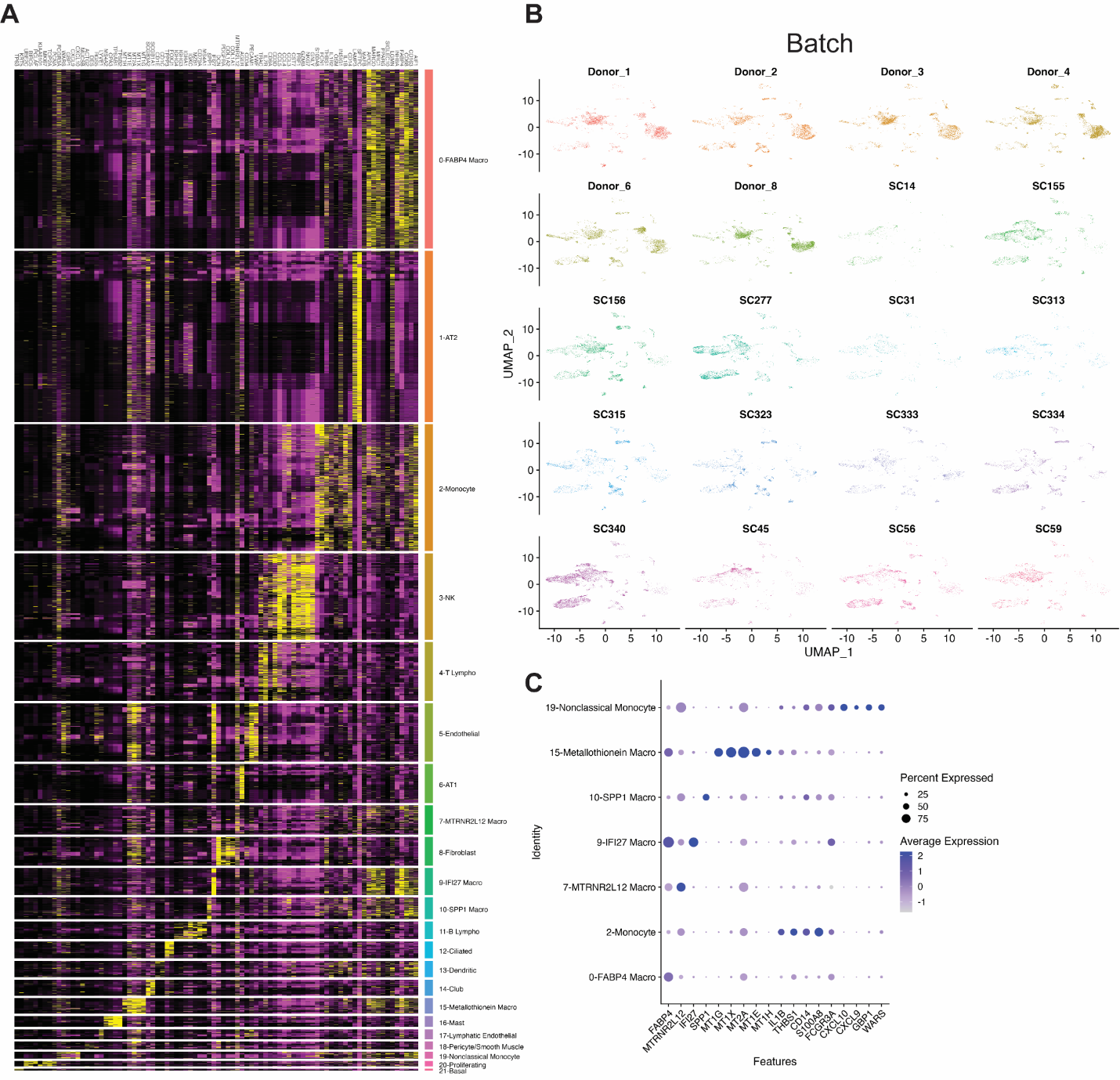


**Supplementary Figure 1.** (A) Heatmap of marker genes for all 22 identified cell types (B) Cells on the UMAP plot of all 29 samples were colored by batch (C) Expression of gene markers for myeloid populations in human lungs.


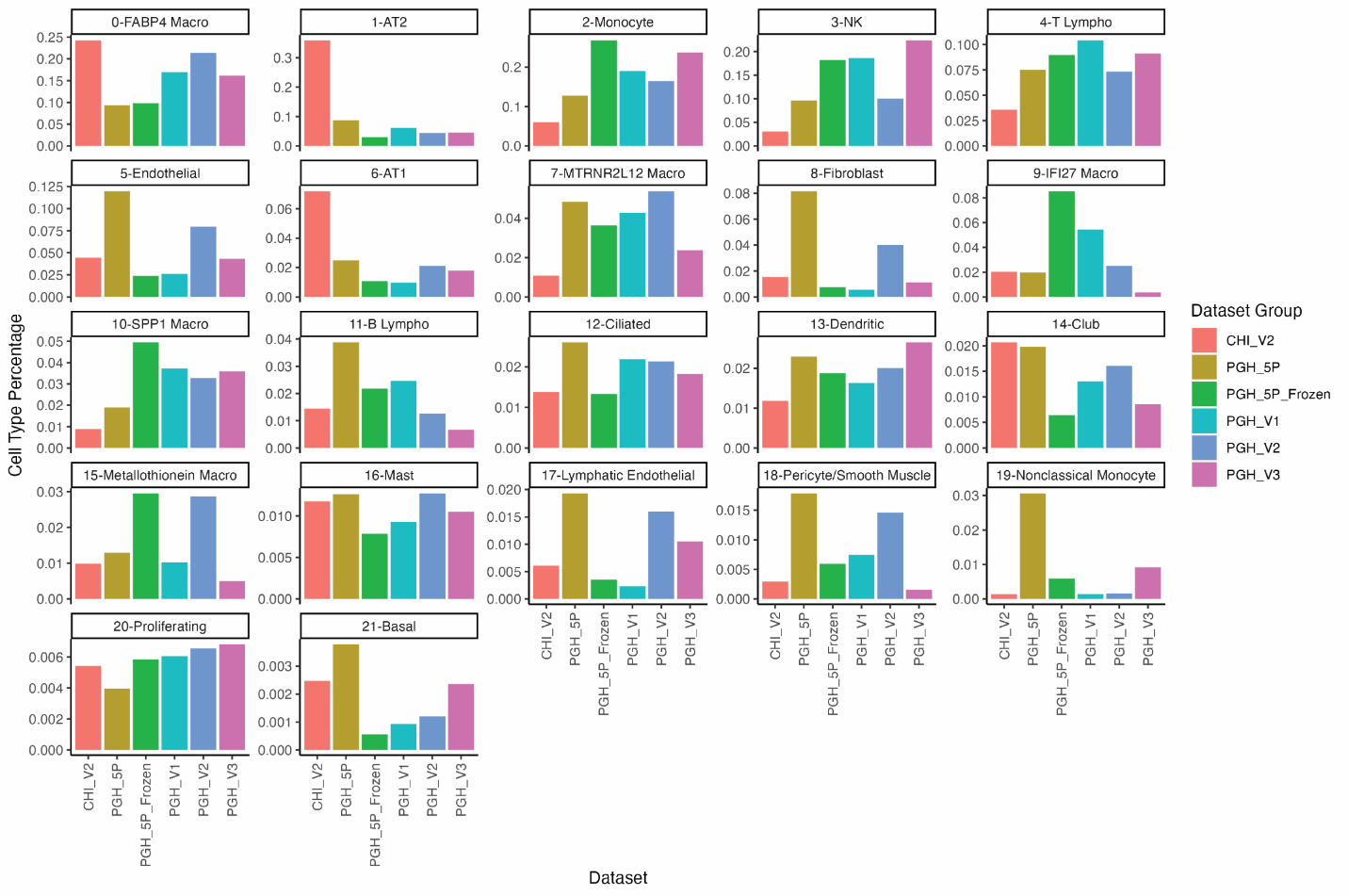


**Supplementary Figure 2.** Barplots showing cell type percentage by dataset group for each cell type.


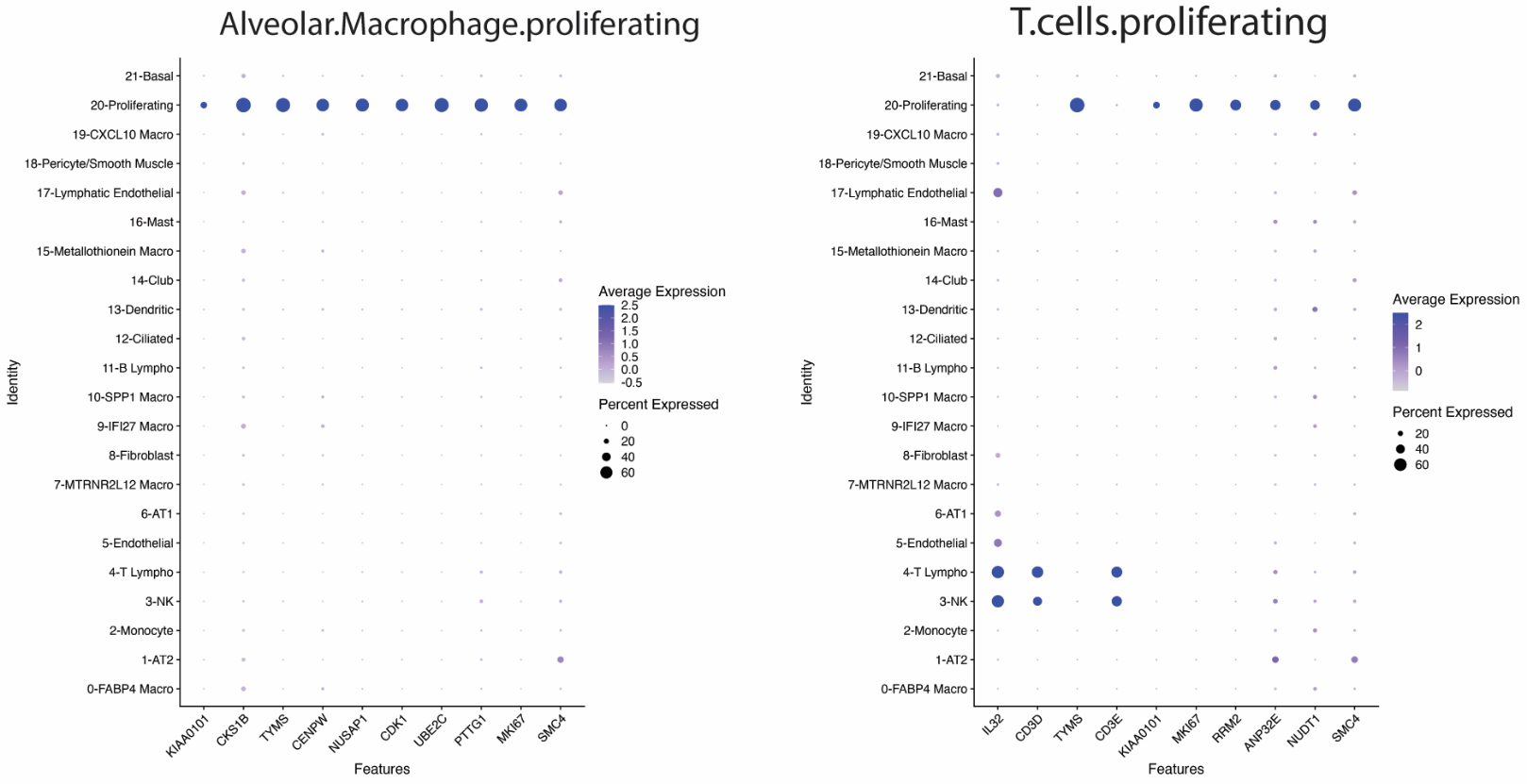
 **Supplementary Figure 3.** Expression of canonical markers for proliferating alveolar macrophage and T cell in human lung.


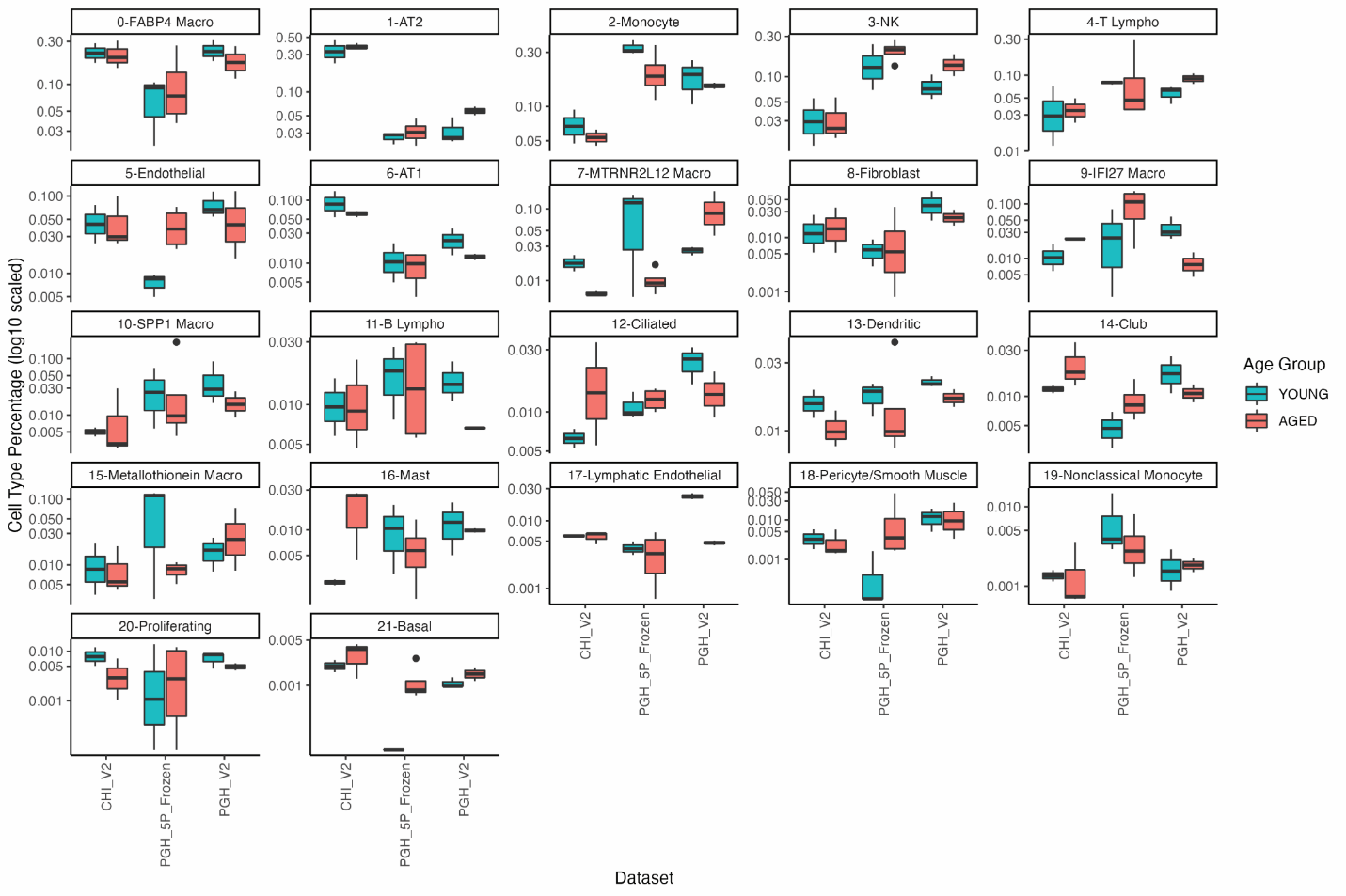


**Supplementary Figure 4.** Boxplots showing cell type percentage for age groups across cell type and data group.


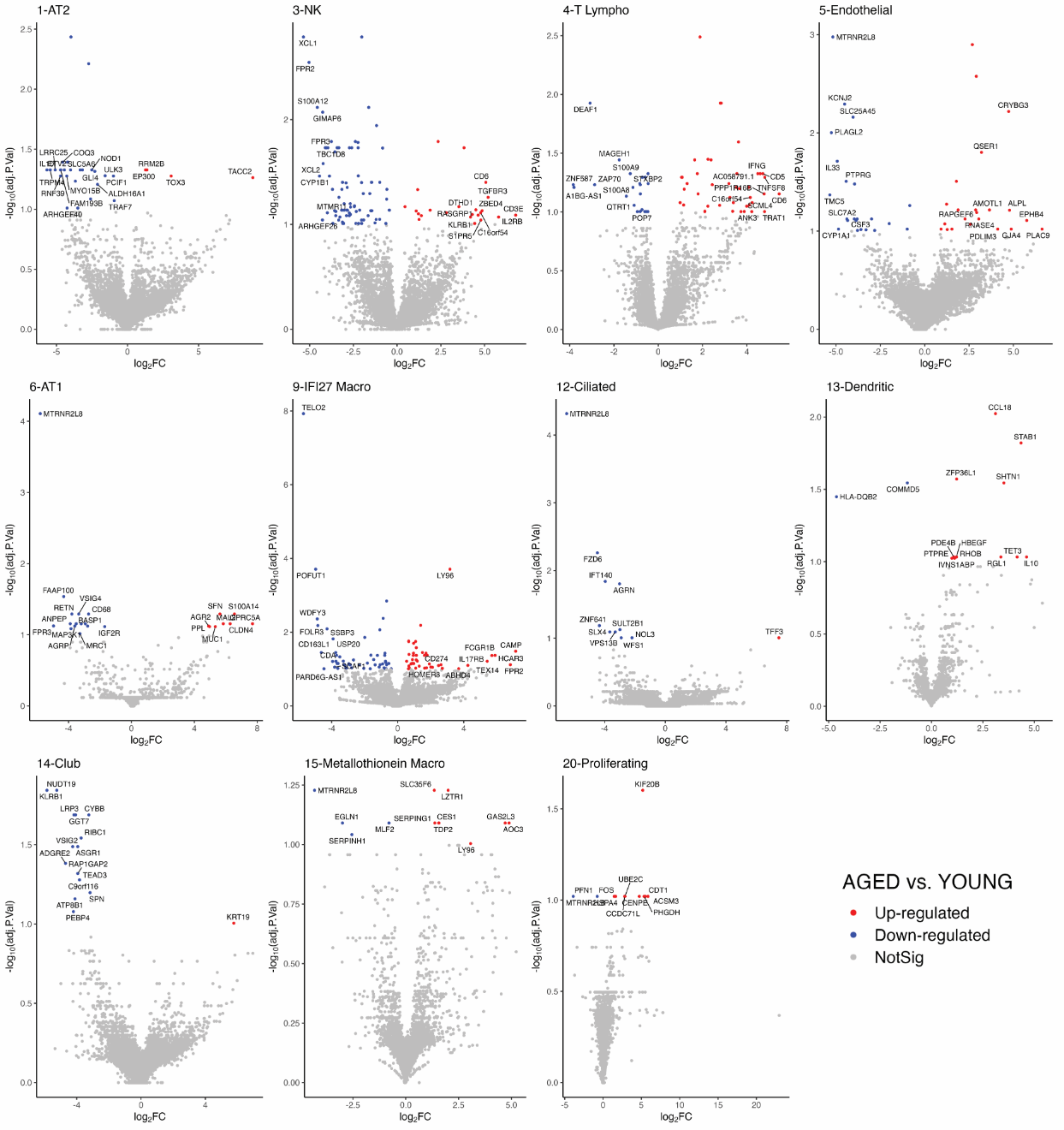
 **Supplementary Figure 5.** Volcano plots showing DEG results across cell types with significant DEGs.


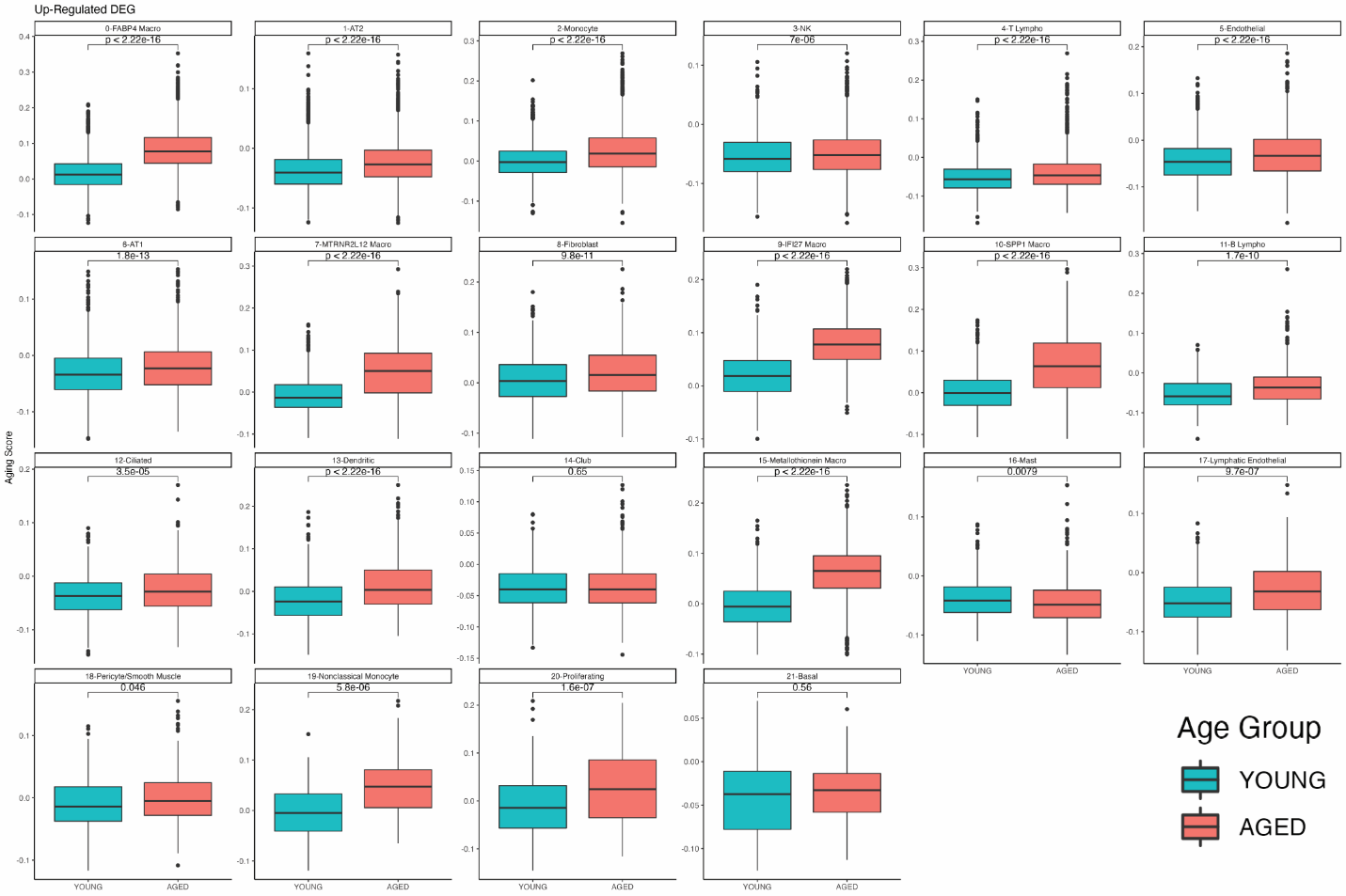
 **Supplementary Figure 6.**  Box plots showing the distribution of aging scores based on up-regulated DEGs on each cellular population (Wilcoxon test).


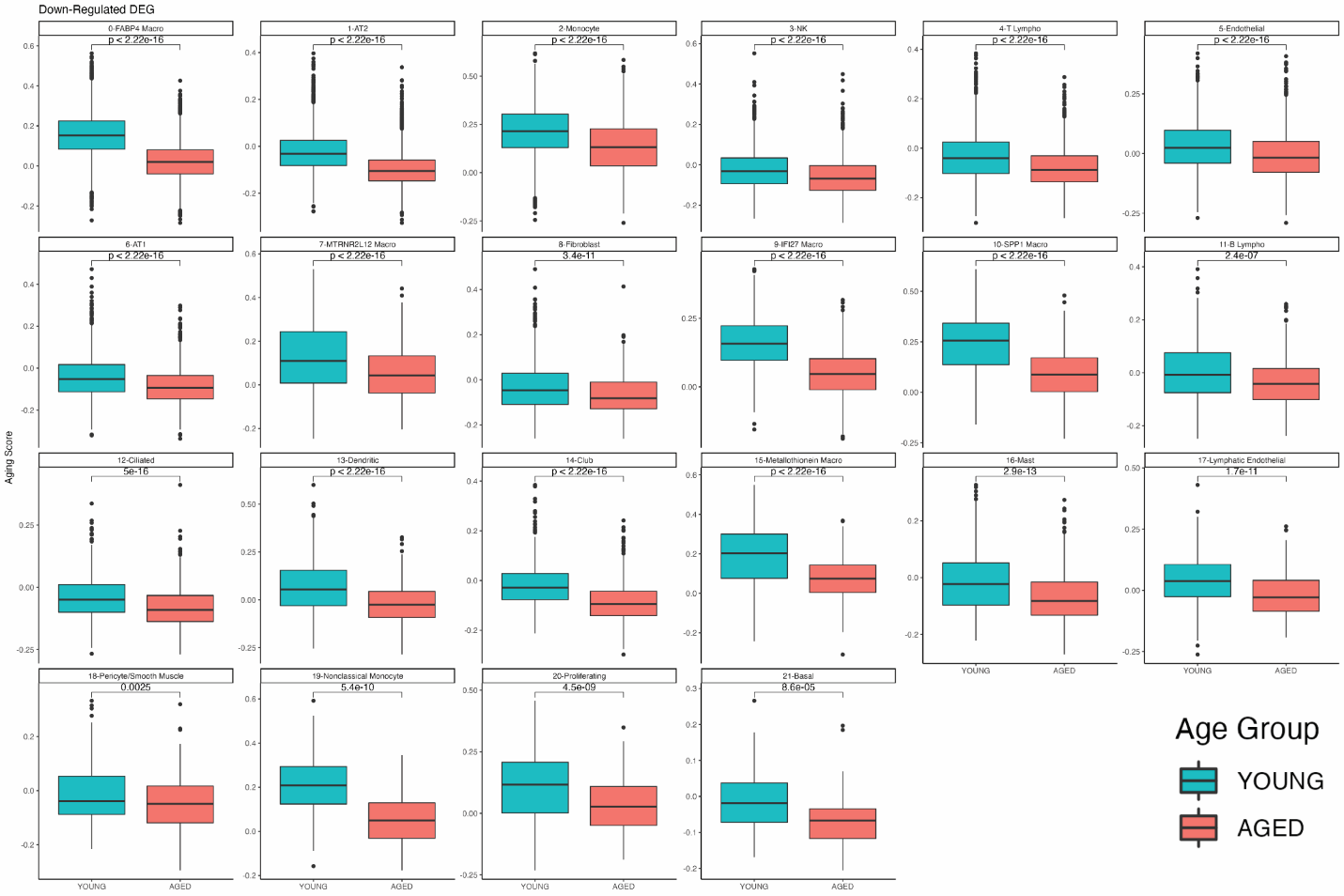
 **Supplementary Figure 7.**  Box plots showing the distribution of aging scores based on down-regulated DEGs on each cellular population (Wilcoxon test).


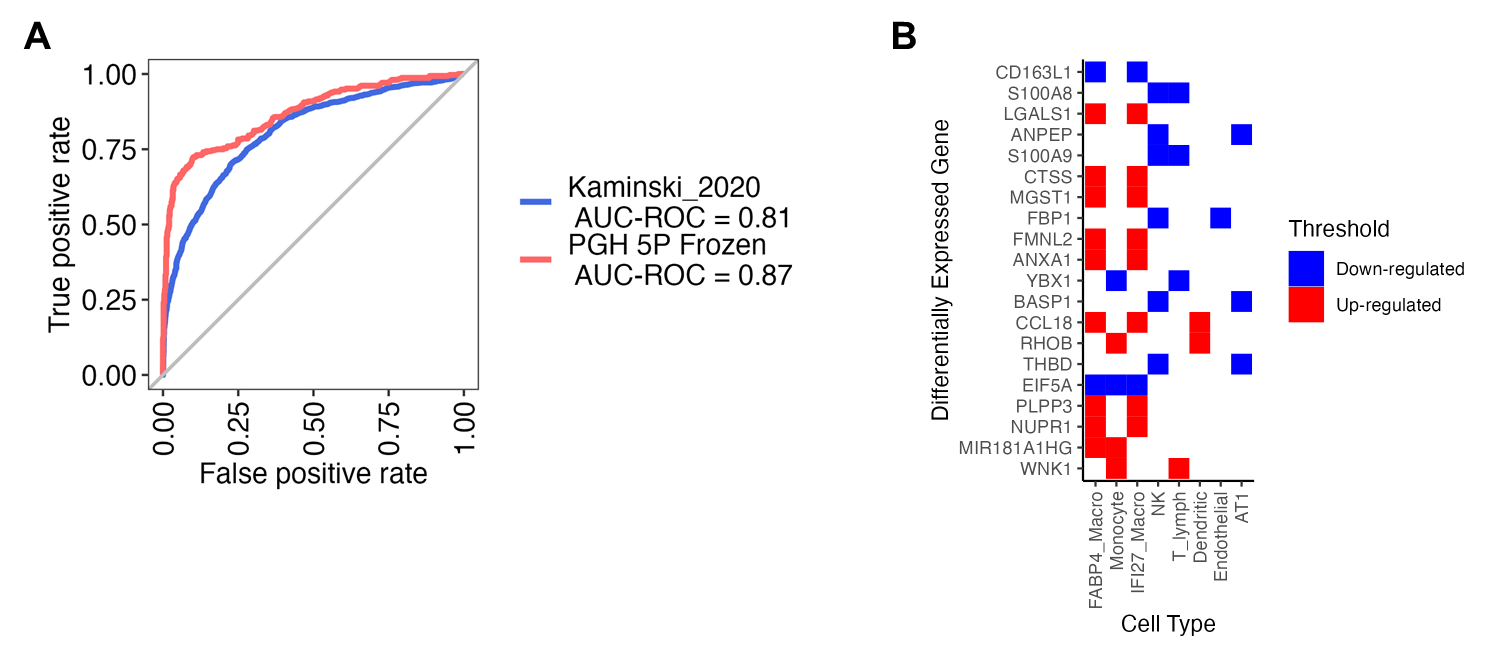


**Supplementary Figure 8. Performance of age state classifier on validation dataset.** (A) The area under the receiver operating characteristic (AUROC) evaluated behavior of the random forest classifier on our own data (Red) and validation set (blue).  (B) Heatmap of the top 20 most important variables in the classification task for the Kaminski data set colored as either up-regulated in red or down-regulated in blue across cell types.


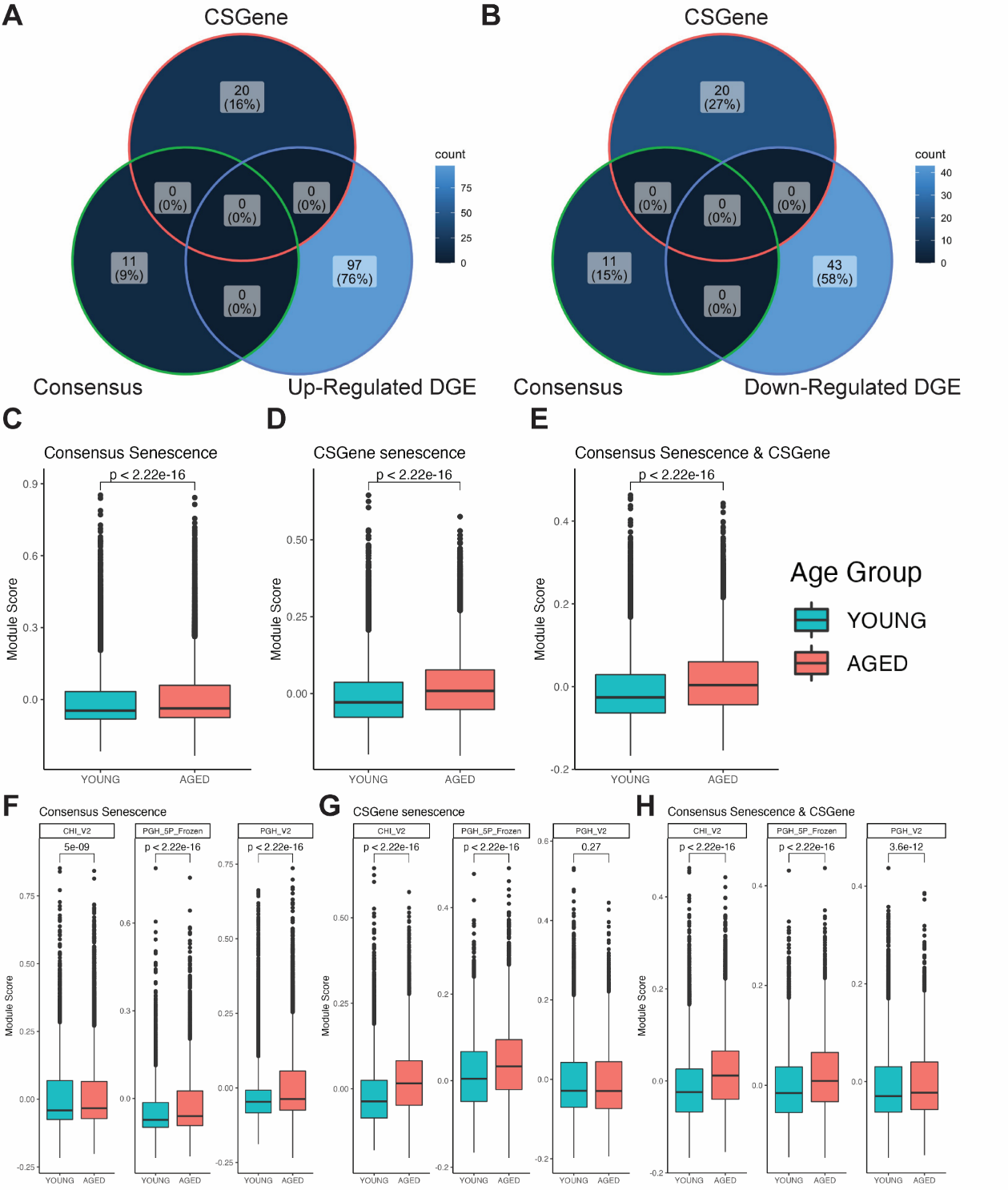


**Supplementary Figure 9. Senescence Score Calculation on Aged Lungs.** (A and B) Venn diagrams showing overlaps among Consensus Senescence gene signature, top 20 ranked CSGene and our up-regulated (A) or down-regulated (B) Lung Aging Gene Signatures. (C, D and E) Box plots showing senescence scores inferred from three senescence gene signatures in all cohorts. (C) 11 Consensus Senescence gene signature, (D) top 20 ranked cell senescence genes (CSGene), (E) union of the two gene sets. (F, G and H) Box plots showing senescence scores inferred from three senescence gene signatures divided by cohort. (F) Consensus Senescence, (G) CSGene, (H) the union of the two.


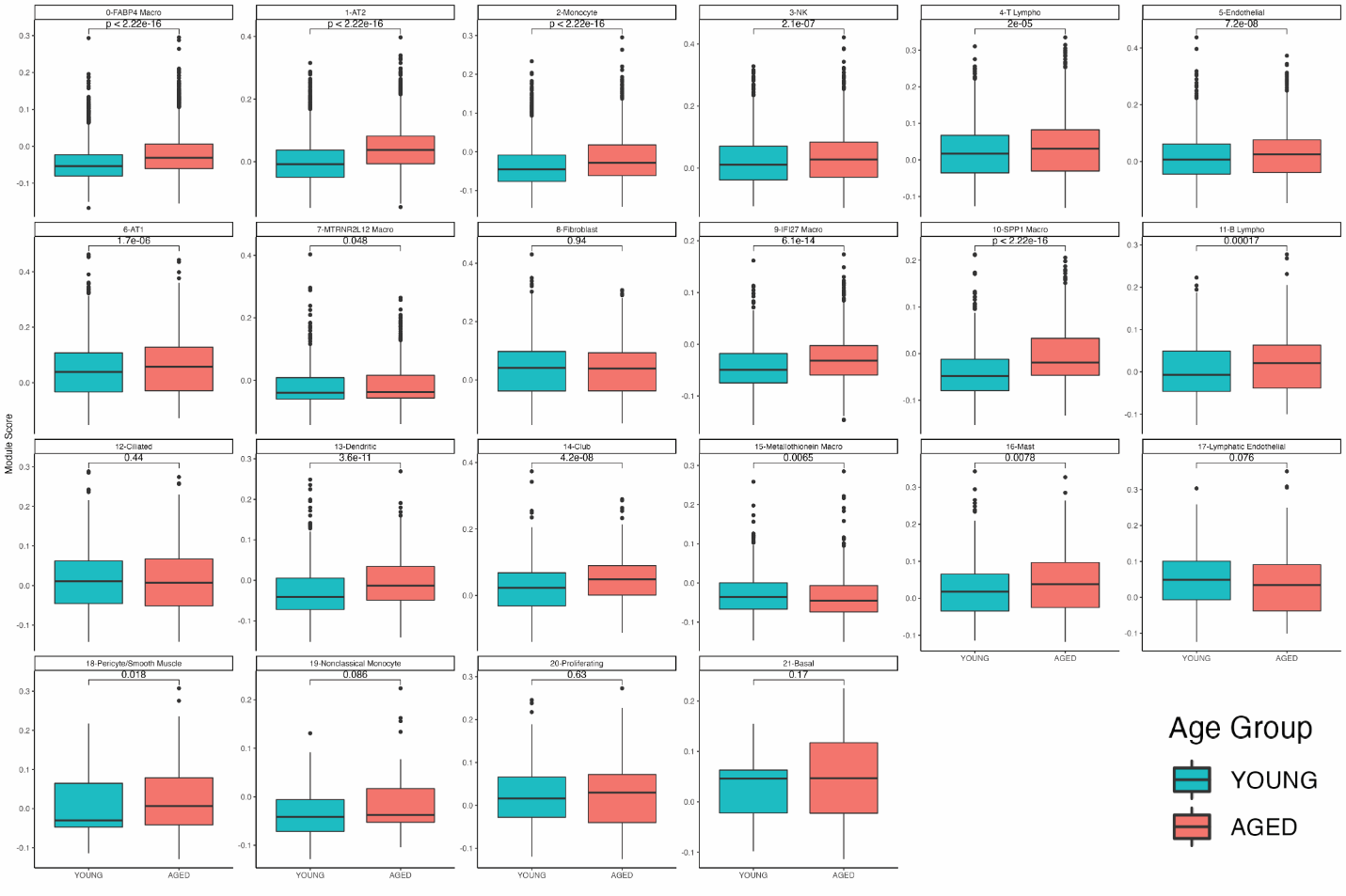
 **Supplementary Figure 10.**  Boxplots of module score distribution of the union of genes in Consensus SEnesence + CSGene gene signatures in cell populations. All cell types are presented, although all but 5 cell types show significant differences in the module score.
